# The dual role of native plant diversity in shaping plant invasions: scale and habitat dependence in urban-rural ecotones

**DOI:** 10.3389/fpls.2026.1786551

**Published:** 2026-03-13

**Authors:** Qiuyan Chen, Huijie Zhao, Pengbo Yan, Lanxi Li, Rongxiao He, Xiaomeng Chen, Fan Yang

**Affiliations:** 1 College of Tropical Agriculture and Forestry, Hainan University, Haikou, China; 2 College of Tourism Ecology and Environment, Guilin Tourism University, Guilin, China; 3 Shan Shui Conservation Center, Beijing, China

**Keywords:** urban-rural ecotones, native herbaceous plants, invasive herbaceous plants, species diversity, functional diversity, phylogenetic diversity

## Abstract

**Introduction:**

Urban–rural ecotones are characterized by high habitat heterogeneity and intense anthropogenic disturbance, are recognized as high-risk areas for alien plant invasions. To explore the differences in diversity patterns between native and invasive herbaceous plants, as well as their relationships, across various habitat types, and to determine the major environmental factors facilitating invasion, we conducted a comprehensive field survey in Jiangdong New District, Haikou City, Hainan Province, a rapidly urbanising ecotone in southern China.

**Methods:**

A total of 537 herbaceous plant quadrats were established across six representative habitat types selected according to *in situ* habitat heterogeneity at 220 sampling sites. We recorded a total of 229 herbaceous plant species, including 155 native and 74 invasive species.

**Results:**

Analyses revealed significant differences in diversity indices and total cover between native and invasive assemblages among habitats. Invasive herbaceous plant diversity was highest in roadside habitat, whereas native herbaceous plants were dominant in landscape green space. Native species diversity was positively associated with invasive species richness, but negatively associated with their total cover and invasion intensity. Suppressive effects of native diversity were strongest in abandoned land and depression wetland habitats. Trampling intensity promoted invasive species richness, whereas proximity to buildings, proximity to water bodies, and higher relative humidity facilitated the formation of high invasive plant cover. In contrast, active management, such as artificial removal, consistently suppressed invasion across all metrics.

**Discussion:**

This study clarifies the scale and habitat-dependent dual role of native herbaceous diversity in regulating invasions, providing scientific support for the development of habitat-targeted management approaches in urban-rural ecotones.

## 1 Introduction

Economic globalization and the increasing frequency of trade activities have accelerated the long-distance dispersal and repeated introduction of invasive species through multiple pathways (Seebens et al., 2021; Potgieter et al., 2024). A variety of local human-driven processes, including urban expansion, transport-network development, and land-use change, have also further facilitated alien plant invasions across diverse habitat (Štajerová et al., 2017; Godefroid and Ricotta, 2018). Alien plants have colonized habitats ranging from minimally disturbed protected areas (Dai et al., 2025) and densely populated urban built-up areas (Boukita et al., 2025) to urban-rural ecotones and predominantly agricultural rural landscapes (Ariori et al., 2017; Chatterjee and Dewanji, 2024). Once established, such species can rapidly colonize and spread in novel environments (Gioria et al., 2023), threatening invaded ecosystems by altering habitat structure and disrupting ecological processes (Jones and McDermott, 2018; Kumar Rai and Singh, 2020), as well as causing substantial economic losses (Bradshaw et al., 2016).

As ecological vulnerable regions, urban-rural ecotones, distinctive transitional zones between urban and rural ecosystems, are strongly shaped by urban expansion (Wandl and Magoni, 2017). Highly complex land-use patterns and intense human disturbances make them hotspots for alien plant invasions (Afonso et al., 2020). From a management perspective, urban-rural ecotones are largely non-built-up areas characterized by fragmented land ownership and frequent disconnects among planning, regulation, and ecological management (Wandl and Magoni, 2017). These issues often lead to weak prevention measures and insufficient removal of invasive species. From an ecological perspective, the diversity of land-use types and varying human activity intensity within ecotones create a unique mosaic of habitats. Microclimatic conditions (Xie et al., 2024), soil properties (Mao et al., 2014), and resource supply patterns (Afonso et al., 2020) in ecotones differ significantly from both urban built-up and rural areas. Landscape complexity and divergent environmental conditions jointly generate high internal habitat heterogeneity, which shapes resource acquisition and competitive interactions to drive pronounced variation in native-invasive plant associations across habitat types.

The heterogeneous habitats of urban-rural ecotones provide a critical context for testing two classic hypotheses regarding how native communities influence invasive species. Historically, the biotic resistance hypothesis posits that communities with higher native species richness are more resistant to alien invasions (Elton, 1958; Cheng et al., 2024), as greater native diversity limits available resources and niches (Dai et al., 2025; Liu et al., 2025b). Conversely, an increasing number of studies indicate that native and invasive richness may also be positively correlated, influenced by factors such as environmental heterogeneity and disturbance intensity (Jauni and Hyvönen, 2012; Kim et al., 2025), supporting the biotic acceptance hypothesis (Stohlgren et al., 2006). Simply assessing these patterns using species richness as a single biodiversity metric (Shi et al., 2025) may limit our understanding of how native community structure and function influence invasion. Consequently, recent research has increasingly adopted metrics such as functional diversity and phylogenetic diversity to capture complementary facets of biodiversity (Ernst et al., 2022). High functional diversity and specific functional-group compositions in native plant communities can enhance biotic resistance to alien species (Byun et al., 2013), while high phylogenetic diversity may confer resistance through increased differentiation (Ernst et al., 2025). However, most studies have been conducted in relatively homogeneous or single ecosystem types. In the urban-rural ecotone with highly heterogeneous landscapes, how “resistance” and “acceptance” effects co-occur across different dimensions remains to be further elucidated.

Located at the rapidly expanding fringe of Haikou City, the Jiangdong New Area is characterized by frequent population movement, a highly heterogeneous mosaic of land-use types, and substantial disturbances driven by urban development, thereby exhibiting the typical features of urban-rural ecotones. These characteristics have led to increasingly prominent issues of invasive alien plants within the area, and the high fragmentation of native vegetation (Cao et al., 2024; He et al., 2024), which may have different impacts on the relationship between native and invasive plants. Therefore, using the Jiangdong New Area as a model system, this study aims to address the following three scientific questions: (1) How do the diversity patterns of invasive and native herbaceous plants differ among various habitat types within urban-rural ecotones? (2) How does the relationship between native herbaceous plant diversity and invasive plants, as measured by different assessment indicators, reflect biotic acceptance and resistance in urban-rural ecotones, and in which habitat types is this relationship most pronounced? (3) Which environmental factors significantly affect the richness, total cover, and invasion intensity of invasive herbaceous plants?

Specifically, we hypothesize that: (A) invasive herbaceous plants exhibit the highest diversity in roadside habitats, whereas native herbaceous plants are dominant in landscape green spaces; (B) the relationship between native and invasive herbaceous plants exhibits a dual role that varies across assessment indicators, reflecting both biotic acceptance and biotic resistance, such that under environmental heterogeneity, higher native plant diversity is positively correlated with invasive species richness but negatively associated with invasive total cover and invasion intensity, and native communities in abandoned farmland and depression wetlands exert stronger inhibitory effects on invasive plants; (C) higher canopy cover, lower proportions of impermeable surfaces, and artificial removal suppress invasion, whereas greater trampling intensity and higher relative humidity facilitate invasion.

## 2 Materials and methods

### 2.1 Study area

Jiangdong New District (110°22′–110°38′E, 19°53′–20°04′N) is located on the eastern coast of Haikou City on Hainan Island, China, covering an area of approximately 298 km². The district lies within the northeastern coastal plain of Hainan Island and is characterized by low and flat terrain. It is bordered by the Nandu River to the west, the Qiongzhou Strait to the north, and the Dongzhai Gang National Nature Reserve to the east. It has a typical tropical monsoon oceanic climate, with a mean annual temperature of 24.3 °C and an average annual precipitation of 2067 mm. The region receives more than 2000 h of sunshine annually.

**Figure 1:**
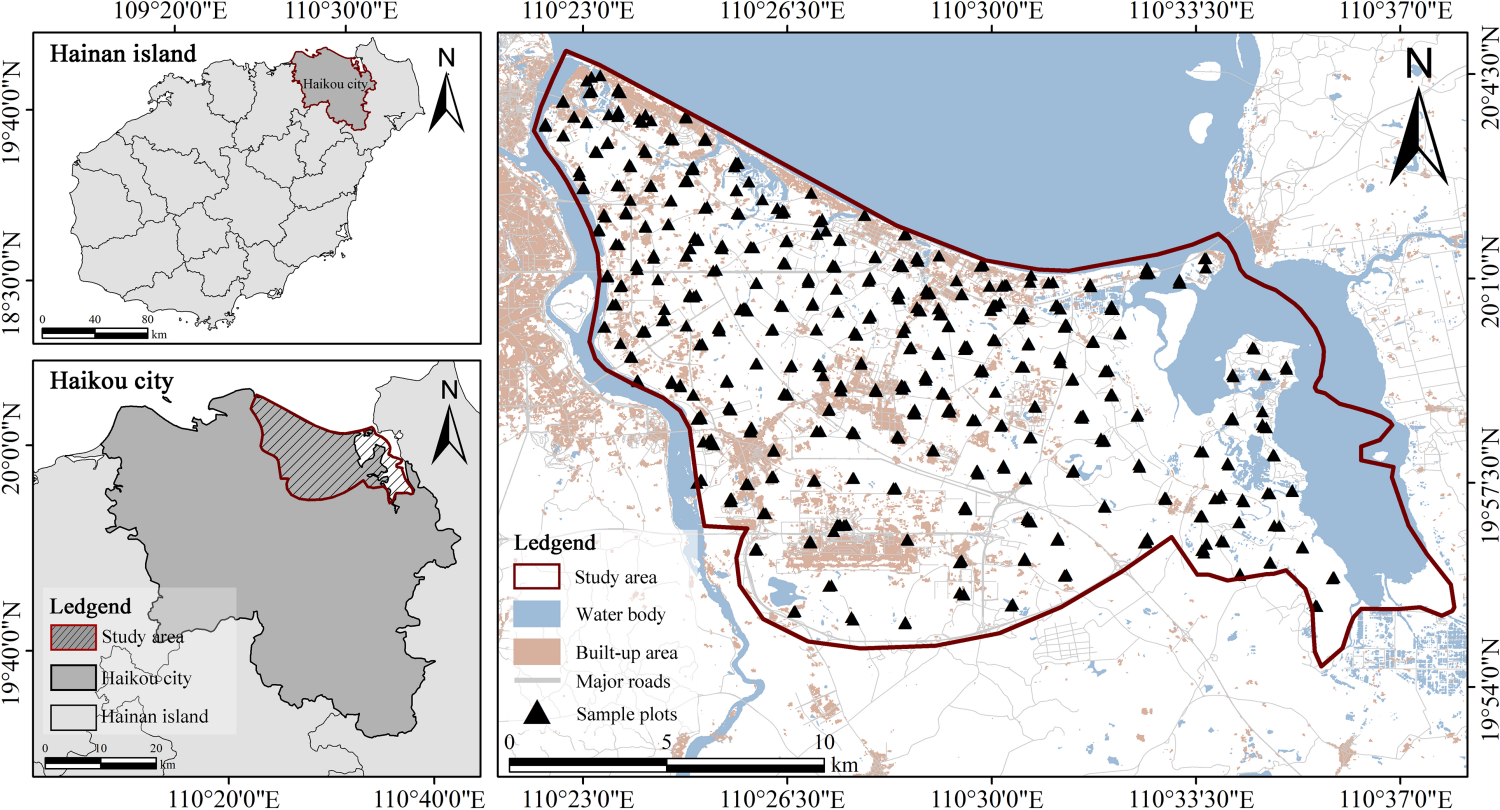
Study area and distribution of sample plots.

Since being incorporated into the China (Hainan) Pilot Free Trade Zone in 2018, Jiangdong New District has gradually developed a spatial pattern characterized by the interspersed distribution of built-up land and natural ecological spaces during the course of regional development and construction. The area is characterized by complex habitat types, frequent anthropogenic disturbances, and relatively weak management, exhibiting typical features of an urban-rural ecotone. Such conditions are conducive to the establishment, reproduction, and spread of invasive herbaceous plants. Consequently, Jiangdong New District provides an ideal study area for investigating the relationships between invasive herbaceous plants and native herbaceous plants across different habitat conditions, as well as their responses to habitat-related environmental factors (**Figure 1**).

### 2.2 Field survey

#### 2.2.1 Sample design

We applied a spatially balanced sampling approach in ArcMap 10.7 (Esri) to select 220 sampling sites in the Jiangdong New District. Based on the environmental characteristics of these sites, we classified all plots into six habitat types to ensure adequate representation of habitat conditions across the study area: roadside habitat (RS), forest edge/understory (FEU), abandoned land (AL), farmland/nursery (FN), depression wetland (DW), and landscape green space (LGS) (**Table 1**). During field surveys, we adjusted the number of sample plots at each site according to local habitat heterogeneity: 1–2 plots were established in areas with relatively homogeneous conditions, while 1–2 plots were set separately for each distinct habitat type in more heterogeneous areas. The differences in quantity distribution across habitats reflected the actual landscape composition and heterogeneity of the rapidly urbanized Jiangdong New Area. We used a size of 4 square meters (2 × 2 m) as the standard plot area. For habitats with linear features, the plot shape was adjusted to 1 × 4 m or 0.5 × 8 m based on environmental conditions. This adjustment better suited the geometric shape of linear habitat features (e.g. roadsides and wetlands), minimizing the inclusion of multiple microhabitats within a single plot and resulting in more representative community samples. Ultimately, we recorded data from 537 herbaceous plant community plots (**Figure 1**).

**Table 1:** Habitat type information table.

Habitat type	Number of plots	Habitat characteristics
Roadside habitat	123	Disturbed areas located along road edges, field ridges, and both sides of transportation corridors.
Forest edge/understory	94	Habitats distributed along the edges of natural secondary forests and beneath the forest canopy.
Abandoned land	142	Bare lands that are idle or abandoned.
Farmland/nursery	59	Agricultural production and seedling cultivation areas, strongly influenced by human management.
Depression wetland	38	Moist habitats in low-lying areas with water accumulation or surrounding water bodies.
Landscape green space	81	Green spaces formed through artificial construction and management, serving both landscaping and recreational functions.

#### 2.2.2 Herbaceous plants survey

Within each plot, we recorded herbaceous plant species composition, coverage, plant height, and individual abundance. Coverage was estimated by measuring the length and width of the boundaries using pre-prepared measuring tapes. Plant height was measured with the assistance of a measuring tape or T-shaped ruler, and the average height of the majority of individuals within the population was taken as the representative height. For sparsely distributed herbaceous populations, the number of individuals was recorded directly, whereas for densely growing populations, species number was estimated using the area occupied by five representative individuals together with the total cover of the species within each quadrat, which was then used to compute relative density of species.

#### 2.2.3 Environmental factor collection

During field surveys, the main habitat factors were recorded for each sample plot, including canopy cover (CC), impervious surfaces (IS), trampling intensity (TI), artificial removal (AR), proximity to buildings (NTB), proximity to roads (NTR), and proximity to water bodies (NTW) (**Table 2**). Meanwhile, the relative humidity (RH) data of the sample sites were obtained from ClimateAP (Wang et al., 2017), which provides site-level climate estimates by integrating geographic variables such as latitude and elevation.

**Table 2:** Description of habitat factors.

Environmental factor (abbreviation)	Variable type	Source	Classification method
Canopy cover (CC)	Ordinal variable	Field sampling	Based on canopy density thresholds reported in earlier studies (Hou et al., 2023) and adjusted to reflect the conditions of the study area, canopy cover was classified into four levels: 0: Non-forested conditions (0–0.2) 1: Sparse cover (0.2–0.39) 2: Moderate cover (0.4–0.69) 3: High cover (0.7–1)
Impervious surfaces (IS)	Dummy variable	Field sampling	Classified according to the surface type based on field observations: 0: Natural surface 1: Impermeable surface
Trampling intensity (TI)	Ordinal variable	Field sampling	Recorded based on site accessibility (Conradi et al., 2015) and categorized into four levels: 3: Located directly on the path (0 m) 2: Situated at the path margin (0.1 m) 1: Positioned 1.5 m from the path edge 0: Located 15 m away from the path
Artificial removal (AR)	Ordinal variable	Field sampling	Classified based on the combined assessment of bare soil proportion and relative plant height (Wang et al., 2021b): 0: Relatively high plant height, low proportion of bare soil 1: Relative plant height and bare soil proportion are moderate 2: Relatively low plant height, high proportion of bare soil
Proximity to buildings (NTB)	Dummy variable	Field sampling	Classified based on field observations of the effective influence ranges of buildings, roads, and water bodies during the survey. Distance thresholds were defined to determine whether sampling plots were directly influenced by built environments or water bodies: 0: No buildings present near the plot (> 20 m) 1: Plot located adjacent to or close to buildings (≤ 20 m)
Proximity to roads (NTR)	Dummy variable	Field sampling	0: Plot located far from roads, not directly disturbed by roads (> 20 m) 1: Plot located adjacent to or close to roads (≤ 20 m)
Proximity to water bodies (NTW)	Dummy variable	Field sampling	0: Plot not close to water bodies (> 10 m) 1: Plot close to water bodies (≤ 10 m)
Relative humidity (RH)	Continuous variable	ClimateAP	Obtained as a continuous variable from ClimateAP data (Wang et al., 2017).

### 2.3 Species information statistics

Species identification was primarily based on the Flora of China (Compilation Committee of the Flora of China, 1959-2004), Flora of Hainan (South China Institute of Botany, Chinese Academy of Sciences, 1964-1977), Inventory of Plant Species Diversity of Hainan (Xing et al., 2012), and Hainan Plant Directory (Yang, 2013). Invasive herbaceous plants were identified according to the Chinese List of Invasive and Naturalized Plants (https://www.cvh.ac.cn/iapc/) and the China Invasive Alien Species Information System (https://www.iplant.cn/ias/). All taxonomic classifications followed the APG IV system proposed by the Angiosperm Phylogeny Group (The Angiosperm Phylogeny Group et al., 2016).

### 2.4 Data processing

#### 2.4.1 Species importance value calculation

To evaluate the relative ecological role of species within the plant community, the species importance value (IV) was calculated using the following formula:


$$IV=\frac{C_{r}+H_{r}+D_{r}}{3}\times 100\%$$

where *IV* is the importance value used to evaluate species dominance in the community; $C_{r}$ represents the relative coverage, indicating the proportion of ground area covered by a species relative to the total coverage by all species; $H_{r}$ denotes the relative plant height, comparing the average height of a species to that of the community; $D_{r}$ is the relative density, reflecting the number of individuals of a species as a percentage of the total number of individuals within the community. The calculations were conducted in R (version 4.2.1) using the “vegan” package (Oksanen et al., 2001).

#### 2.4.2 Invasion degree of invasive herbaceous plants calculation

##### 2.4.2.1 The total cover of invasive herbaceous plants

The total cover refers to the sum of the cover values of all invasive species recorded in a sample plot.

##### 2.4.2.2 Invasion intensity index

The invasion intensity index (III) measured the invasion intensity of a invasive plant within a given plot. We characterized the relative abundance of invasive species by summing up the importance values of all invasive plants in a specific plot, aiming to comprehensively evaluate the dominance and degree of invasion of invasive herbaceous plants in that area. Specifically, a higher invasion intensity indicated that the relative abundance of the invasive species in the plot was closer to its maximum relative abundance across all plots, reflecting a stronger degree of invasion within that plot (Wang et al., 2021a). The calculation method for the invasion intensity index was as follows:


$$III=P_{i}/MaxP_{I}$$

where $MaxP_{i}$ denotes the highest relative abundance of invasive herbaceous species observed across all sampled plots; $P_{i}$ represents the relative abundance of invasive herbaceous species in an individual plot, and larger values of *III* indicate a stronger degree of invasion by invasive herbaceous plants.

#### 2.4.3 Herbaceous plant diversity calculation

Plant diversity was assessed using three complementary dimensions: species, functional, and phylogenetic diversity. Species diversity was characterized by the Patrick richness index (R), the Shannon–Wiener index (H), and the Pielou evenness index (J), using the “diversity” function of the R package “vegan” (Oksanen et al., 2001). Functional diversity was evaluated based on seven plant functional traits related to herbaceous growth, including life cycle, dormancy type, growth form, reproductive strategy, dispersal mechanism, fruit type, and phenological period (**Supplementary Table 1**). All traits are categorical and were used as factor variables to construct a Gower distance matrix, and functional diversity was characterized using functional richness (FRic), functional dispersion (FDis), and Rao’s quadratic entropy (Rao’s Q), which were calculated with the “dbFD” function of the R package “FD” (Laliberté et al., 2009). Phylogenetic diversity was described using Faith’s phylogenetic diversity (PD), mean phylogenetic distance (MPD), and the net relatedness index (NRI). We then used the “V.Phylomaker” package in R to generate our phylogenetic tree and calculate phylogenetic metrics using the “pd” function of the R package “picante” (Kembel et al., 2010).

### 2.5 Statistical analysis

#### 2.5.1 One-way analysis of variance

We used one-way analysis of variance (ANOVA) to compare species richness, the Shannon-Wiener diversity index, functional richness (FRic), functional dispersion (FDis), Faith’s phylogenetic diversity (PD), mean phylogenetic distance (MPD), and total cover of native and invasive herbaceous plants across different habitat types. Tukey’s HSD *post hoc* analysis was used to identify significant differences among habitat types, with distinct uppercase letters denoting statistically different groups (P< 0.05). Subsequently, independent-sample t tests were performed within each habitat to compare native and invasive herbaceous species, and significant differences between the two groups in the same habitat were indicated by different lowercase letters (P< 0.05).

#### 2.5.2 Correlation analysis

We performed Spearman’s correlation analyses to examine the relationships between species richness, total cover, and invasion intensity of invasive herbaceous plants and the species, functional, and phylogenetic diversity of native herbaceous plants, and generated scatter plots with correlation coefficients and significance levels annotated. Based on this analysis, Spearman’s correlation analyses were further conducted across the six habitat types to test these relationships. We used multiple comparison correction to adjust the p-values of these analyses for false discovery rate (FDR). Significant correlations with p-values greater than 0.05 after correction were excluded from the results.

#### 2.5.3 Multiple regression analysis

We collected habitat factor information as predictor variables and processed and transformed them into ordinal and dummy variables suitable for regression analysis (**Table 2**). In constructing the multiple linear regression model, all predictor variables were input as numerical values. The binary variables (IS, NTB, NTR, NTW) were kept in their 0/1 dummy variable encoding. For the ordinal categorical variables (CC, TI, AR), we directly incorporated the recorded rank scores from the survey (**Table 2**) as continuous numerical variables. This meant we assumed that the effect of each level of these ordinal variables on the response variable was linear and equidistant. Although this method simplified the model and helped explain the results, we remained cautious when interpreting the regression coefficients of these variables, focusing on the direction and relative importance of their impact, rather than overinterpreting the precise numerical differences between levels. The richness of invasive herbaceous plants (a count variable), along with the continuous variables of total coverage and invasion intensity, were used as response variables. These response variables were not transformed, as arbitrary transformation may change the target estimate and bias the interpretation of effect size (Schmidt and Finan, 2018). Therefore, to maintain interpretability and because no significant model specification errors were found during diagnostic checks, we retained linear regression for all response variables.

Before fitting the model, we evaluated the multicollinearity among the predictor variables using the variance inflation factor (VIF). All variables showed low collinearity (VIF< 2). We also performed diagnostic checks on the residuals, including Q-Q plots and residual-versus-fitted plots, to assess normality and homoscedasticity. Although slight deviations from normality were observed, linear regression parameter estimation does not require errors to follow a normal distribution; violating the normality assumption mainly affects inference in small samples. With a sufficiently large sample size, the sampling distribution of regression coefficients approximates a normal distribution according to the central limit theorem. Previous studies have shown that linear regression is robust to non−normal residuals (Pek et al., 2018; Schmidt and Finan, 2018). We then fitted a linear regression model (Fox et al., 2001) and tested the spatial autocorrelation of the residuals using Moran’s I. The result indicated weak but significant spatial autocorrelation (Moran’s I = 0.12). Although the residuals exhibited weak spatial autocorrelation, its low magnitude suggests that spatial dependence is unlikely to introduce substantial bias into the main conclusions. All analyses were conducted using R software (version 4.4.3).

## 3 Results

### 3.1 Family and genus composition, diversity and coverage of herbaceous plants

**Figure 2:**
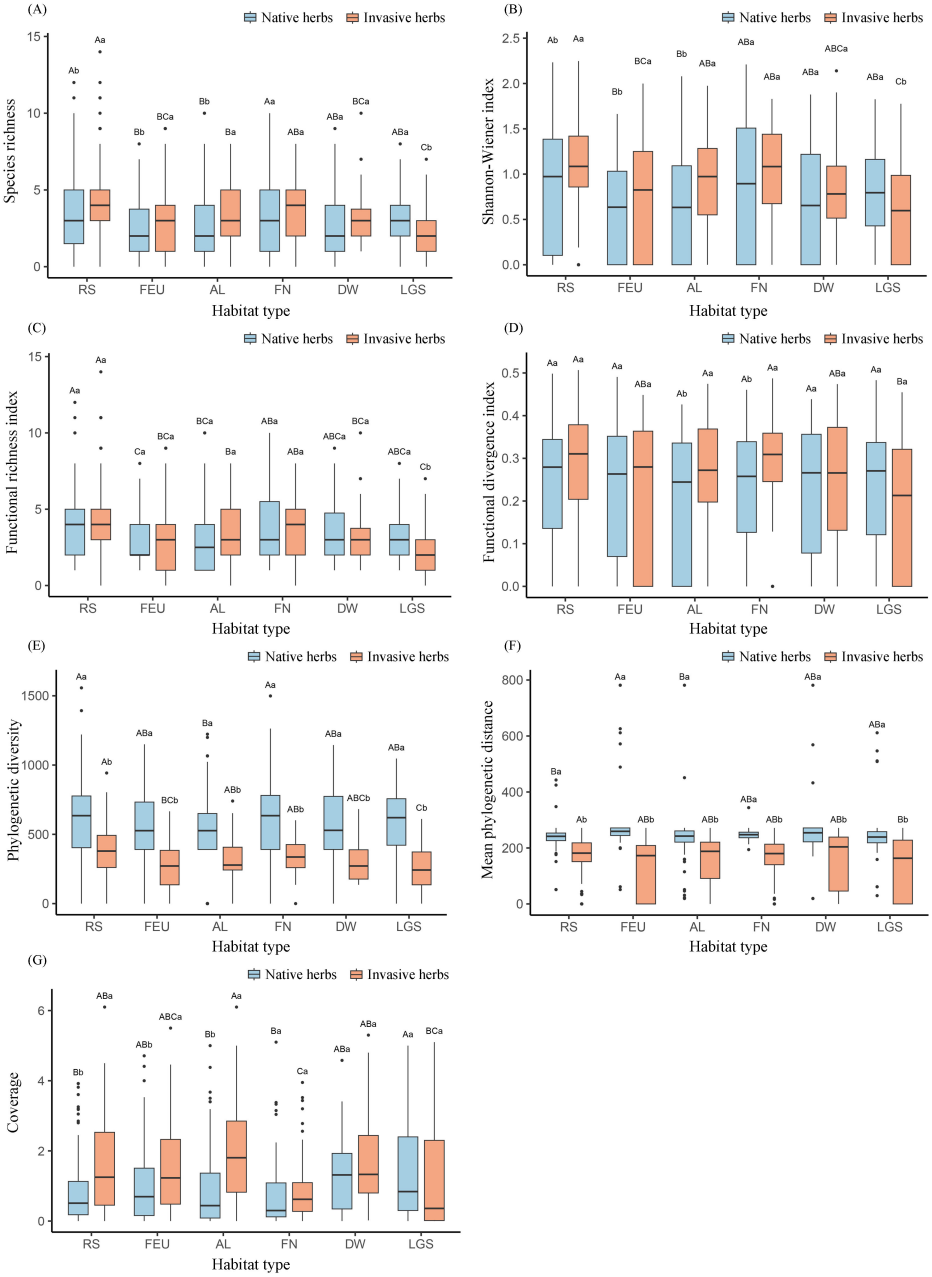
Diversity indices of invasive and native herbaceous plants in different habitats. **(A)** Species richness, **(B)** Shannon-Wiener index, **(C)** Functional richness, **(D)** Functional divergence, **(E)** Phylogenetic diversity, **(F)** Mean phylogenetic distance, and **(G)** Coverage. Identical uppercase letters indicate no significant differences among habitats (P ≥ 0.05), whereas different lowercase letters indicate significant differences between invasive and native herbaceous plants within the same habitat (P < 0.05). RS, Roadside habitat; FEU, Forest edge/understory; AL, Abandoned land; FN, Farmland/nursery; DW, Depression wetland; LGS, Landscape green space.

A total of 229 herbaceous plant species were recorded in this study, comprising 155 native herbaceous plant species from 47 families and 121 genera, and 74 invasive herbaceous plant species from 26 families and 61 genera (**Supplementary Table 2**). One-way analysis of variance revealed significant differences in all diversity metrics and total cover between invasive and native herbaceous plants across habitat types (P< 0.05). For invasive herbaceous plants, roadside habitat showed the highest species richness, Shannon–Wiener index, functional richness, Faith’s phylogenetic diversity, and mean phylogenetic distance (**Supplementary Table 3**; **Figures 2A–F**), whereas the highest total cover occurred in abandoned land (**Figure 2G**).

Among native herbaceous plants, all indices except functional dispersion varied significantly among habitat types (P< 0.05). Shannon–Wiener diversity and functional richness peaked in roadside habitat and were significantly higher than those in forest edge/understory and abandoned land (**Supplementary Table 3**; **Figures 2B, C**). Species richness and Faith’s phylogenetic diversity were significantly higher in roadside habitat and farmland/nursery than in abandoned land (**Figures 2A, E**), while forest edge/understory exhibited the highest mean phylogenetic distance (**Figure 2F**). Native herbaceous plant cover was greatest in landscape green space (**Figure 2G**).

Independent-samples t-tests further showed that, in roadside habitat, forest edge/understory, and abandoned land, invasive herbaceous plants had significantly higher species richness, Shannon–Wiener diversity, and total cover than native herbaceous plants. In contrast, native herbaceous plants dominated these metrics in landscape green space. Functional richness of native herbaceous plants was significantly higher than that of invasive herbaceous plants in landscape green space, whereas functional dispersion of invasive herbaceous plants exceeded that of native herbaceous plants in abandoned land and farmland/nursery. Across all habitat types, native herbaceous plants consistently exhibited higher Faith’s phylogenetic diversity and mean phylogenetic distance than invasive herbaceous plants (**Figures 2A–G**).

### 3.2 Correlation analysis between invasion and native herbaceous plant diversity

#### 3.2.1 Species diversity

**Figure 3:**
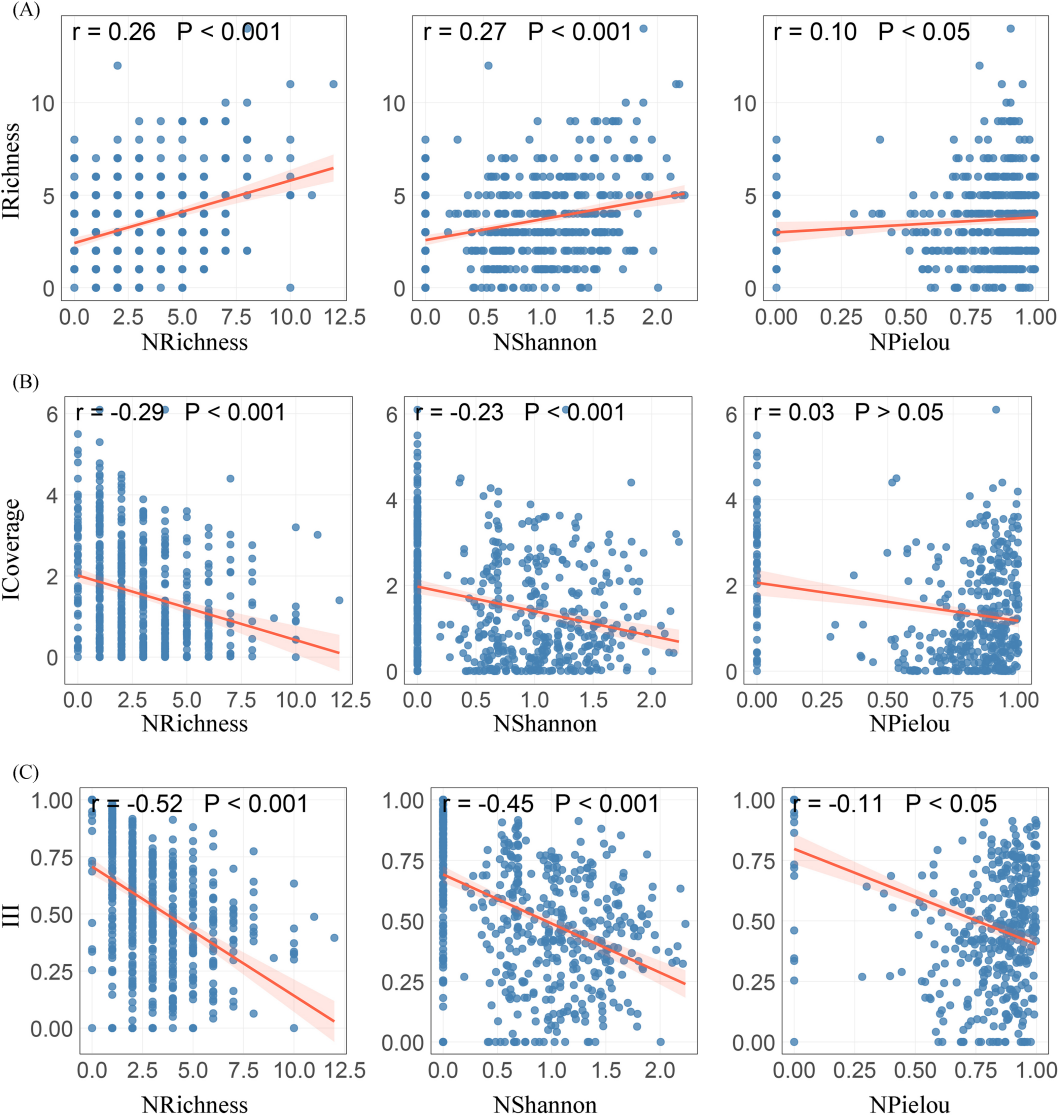
Relationships between native herbaceous plant species diversity and **(A)** invasive richness, **(B)** invasive total coverage, and **(C)** invasion intensity index. IRichness, invasive richness; ICoverage, invasive total coverage; III, Invasion intensity index; NRichness, native species richness; NShannon, native Shannon-Wiener index; NPielou, native Pielou evenness index.

As assumed, when selecting the species diversity of native herbaceous plants as the evaluation indicator, species richness (r = 0.26, P< 0.001), Shannon-Wiener index (r = 0.27, P< 0.001), and Pielou evenness index (r = 0.10, P< 0.05) were all positively correlated with invasive herbaceous plant richness (**Figure 3A**), and this correlation was particularly significant in roadside and forest edge/understory (**Supplementary Figure 1**). In contrast, species richness (r = −0.29, P< 0.001) and Shannon-Wiener index (r = −0.23, P< 0.001) were negatively correlated with total cover of invasive herbaceous plants (**Figure 3B**). This association was particularly significant in abandoned land and depression wetland. Furthermore, species richness (r = −0.52, P< 0.001), Shannon-Wiener index (r = −0.45, P< 0.001), and Pielou evenness index (r = −0.11, P< 0.05) were significantly negatively correlated with invasion intensity (**Figure 3C**). This relationship was significantly observed in all habitat types except for farmland/nursery and landscape green spaces (P<0.05). Indicating that higher native herbaceous plant species diversity can promote the richness of invasive herbaceous plants, but at the same time can suppress the total coverage and invasion intensity.

#### 3.2.2 Functional diversity

**Figure 4:**
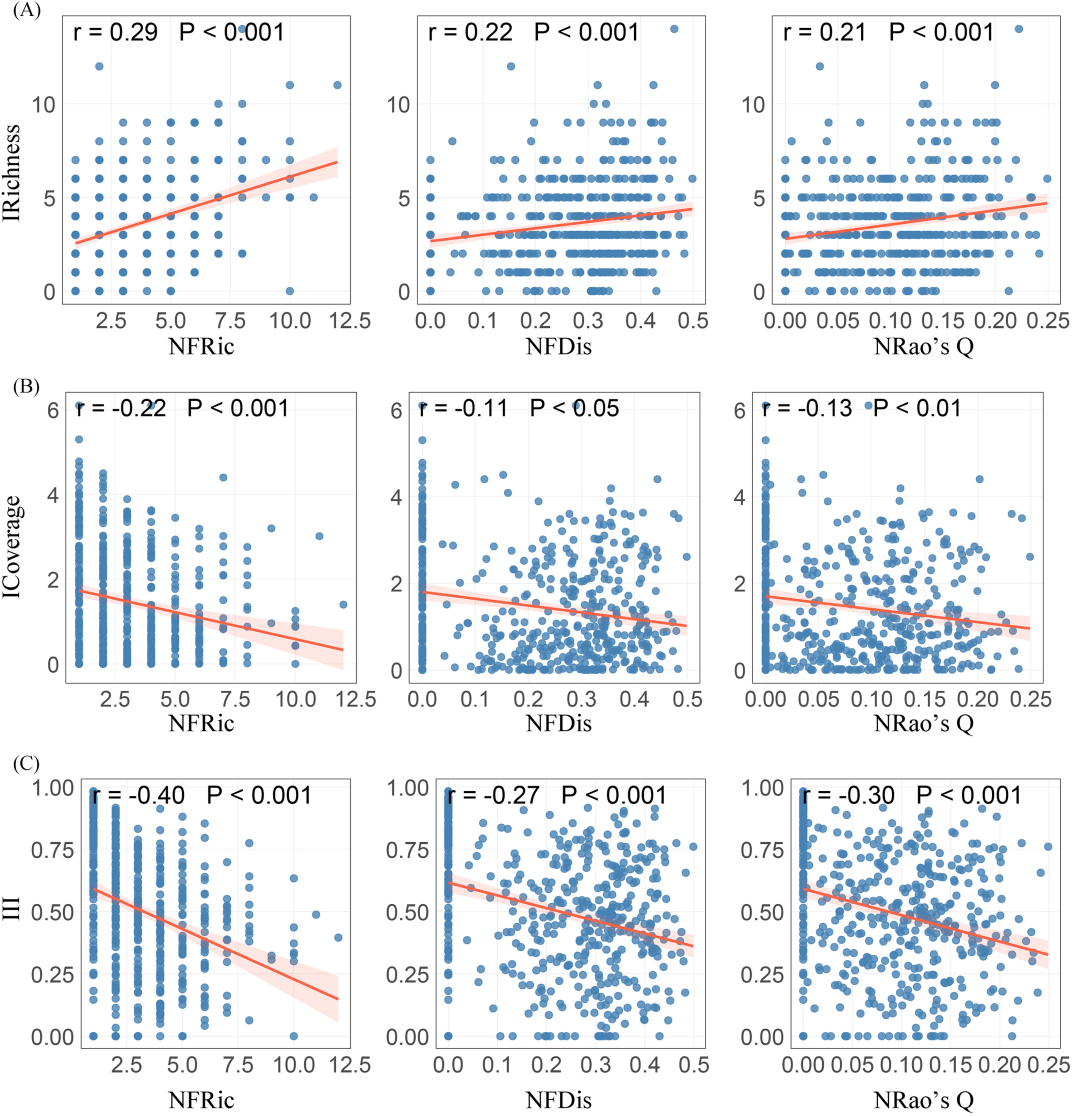
Relationships between native herbaceous plant functional diversity and **(A)** invasive richness, **(B)** invasive total coverage, and **(C)** invasion intensity index. IRichness, invasive richness; ICoverage, invasive total coverage; III, Invasion intensity index; NFRic, native functional richness; NFDis, native functional dispersion index; NRao’s Q, native Rao’s quadratic entropy index.

When using the functional diversity of native herbaceous plants as an assessment indicator, functional richness (r = 0.29, P< 0.001), functional dispersion index (r = 0.22, P< 0.001), and Rao’s quadratic entropy index (r = 0.21, P< 0.001) were significantly positively correlated with the richness of invasive herbaceous plants (**Figure 4A**), and this correlation was particularly significant in roadside, forest edge/understory habitats and landscape green space (**Supplementary Figure 1**). Conversely, all functional diversity assessment indicators were significantly negatively correlated with the total cover and invasion intensity of invasive herbaceous plants (**Figures 4B, C**). The negative correlation with total cover was particularly significant in abandoned land (P< 0.001). The relationship with invasion intensity was most significant in roadside and abandoned land (P< 0.05). This suggests that higher functional diversity of native herbaceous plants can promote the richness of invasive herbaceous plants, while also inhibiting the total cover and invasion intensity.

#### 3.2.3 Phylogenetic diversity

**Figure 5:**
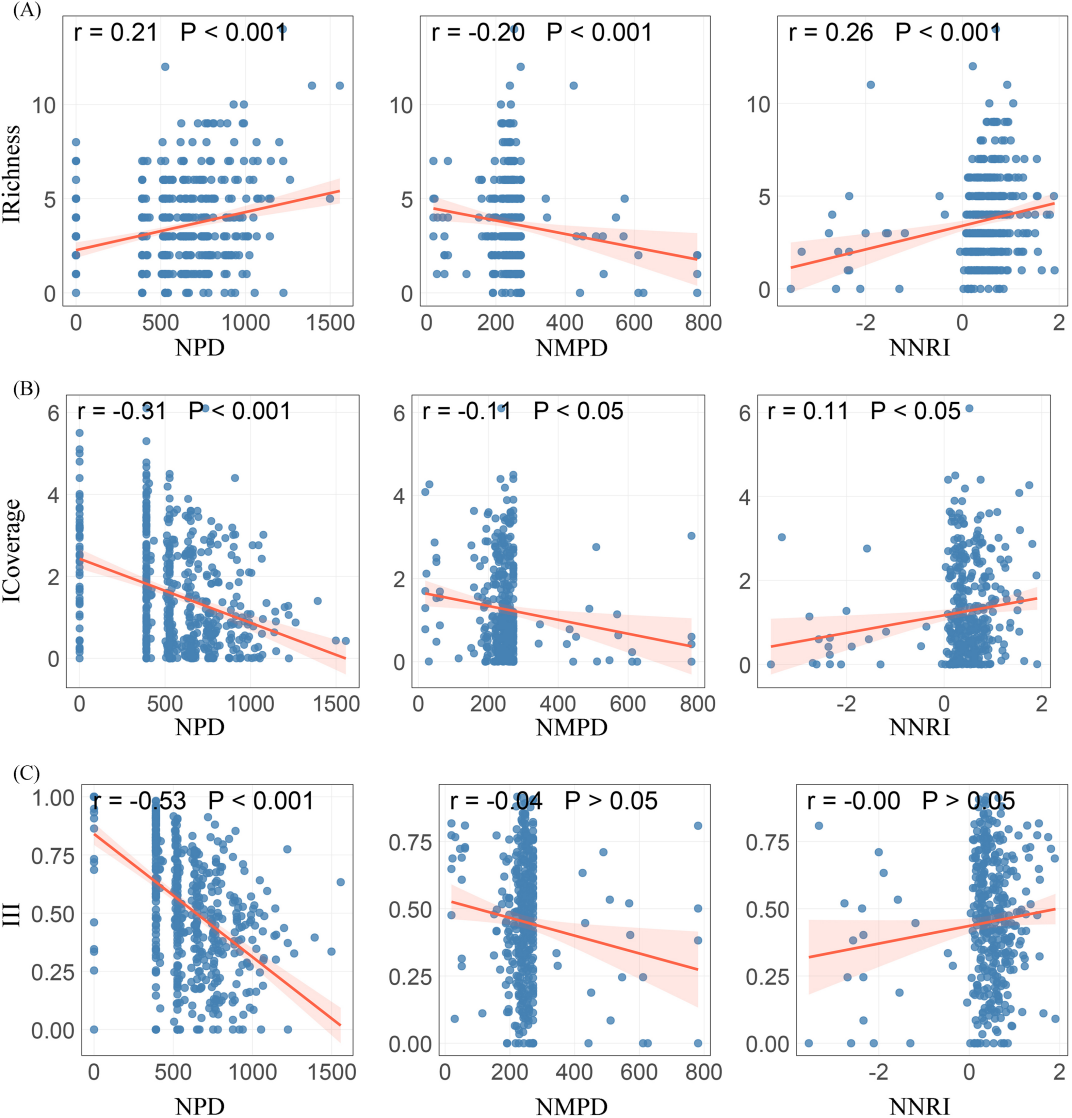
Relationships between native herbaceous plant phylogenetic diversity and **(A)** invasive richness, **(B)** invasive total coverage, and **(C)** invasion intensity index. IRichness, invasive richness; ICoverage, invasive total coverage; III, Invasion intensity index; NPD, native Faith’s phylogenetic diversity; NMPD, native mean phylogenetic distance; NNRI, native net relatedness index.

In terms of the phylogenetic diversity indicators of native herbaceous plants, Faith’s phylogenetic diversity (r = 0.21, P< 0.001) and the net relatedness index (r = 0.26, P< 0.001) were significantly positively correlated with the richness of invasive herbaceous plants (**Figure 5A**). The positive relationship with Faith’s phylogenetic diversity was mainly observed in roadside habitat (P< 0.001), whereas the association with the net relatedness index occurred primarily in forest edge/understory (**Supplementary Figure 1**). Conversely, mean phylogenetic distance (r = −0.20, P< 0.001) was significantly negatively correlated with the richness of invasive herbaceous plants, and this correlation was limited to forest edge/understory. Both Faith’s phylogenetic diversity (r = −0.31, P< 0.001) and mean phylogenetic distance (r = −0.11, P< 0.05) were negatively correlated with the total cover of invasive herbaceous plants (**Figure 5B**). The negative correlation with Faith’s phylogenetic diversity was mainly observed in forest edge/understory, abandoned farmland, and depression wetland (P<0.05), while the negative correlation with mean phylogenetic distance was only observed in forest edge/understory. Faith’s phylogenetic diversity (r = −0.53, P< 0.001) was significantly negatively correlated with invasion intensity (**Figure 5C**). This relationship was observed in all habitat types except farmland/nursery. This indicates that higher phylogenetic diversity and greater phylogenetic dispersion suppress the total cover and invasion intensity of invasive herbaceous plants, while stronger phylogenetic clustering promotes the richness and total cover.

### 3.3 Multiple regression analysis of environmental factors and invasive herbaceous plants richness, total cover and invasion intensity

**Figure 6:**
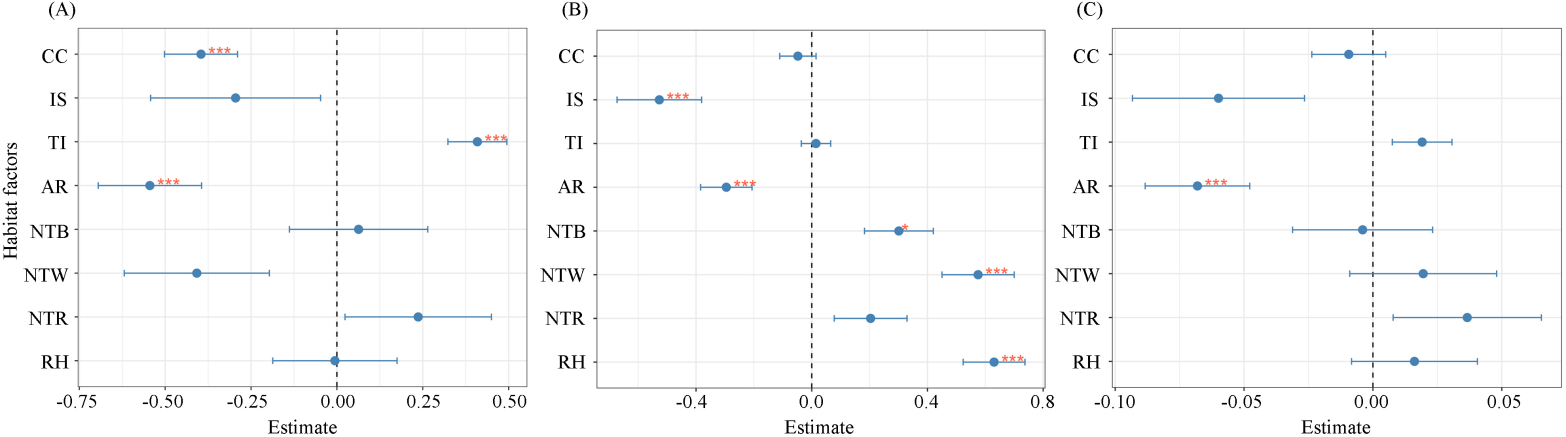
Forest plots illustrating the standardized regression coefficients for habitat factors associated with **(A)** invasive richness, **(B)** invasive total coverage, and **(C)** Invasion intensity index. Note:points represent coefficient estimates (β), error bars denote standard errors, and the vertical dashed line indicates a null effect. Significance levels are shown as ***P<0.001, **P<0.01, *P<0.05. CC, Canopy cover; IS, Impervious surface; TI, Trampling intensity; AR, Artificial removal; NTB, Proximity to buildings; NTW, Proximity to water bodies; NTR, Proximity to roads; RH, Relative humidity.

The results of the multiple regression analysis confirmed hypothesis (C) that environmental factors had significantly different effects on species richness, total cover, and invasion intensity of invasive herbaceous plants (**Figure 6**). Among the environmental factors influencing species richness of invasive herbaceous plants (**Figure 6A**), trampling intensity showed a significant positive association (β = 0.41, P< 0.001), whereas canopy cover (β = −0.396, P< 0.001), and artificial removal (β = −0.545, P< 0.001) were significantly negatively associated with invasive herbaceous plant species richness. With respect to total cover of invasive herbaceous plants (**Figure 6B**), proximity to buildings (β = 0.301, P< 0.05), proximity to water bodies (β = 0.575, P< 0.001), and relative humidity (β = 0.63, P< 0.001) were significantly positively correlated with total cover. In contrast, impervious surface (β = −0.527, P< 0.001) and artificial removal (β = −0.295, P< 0.001) showed significant negative associations with invasive herbaceous plant total cover. Regarding invasion intensity (**Figure 6C**), only artificial removal exhibited a negative effect on invasion intensity (β = −0.068, P< 0.001).

## 4 Discussion

### 4.1 Differential diversity patterns of native and invasive herbaceous plants among habitat types in urban-rural ecotones

This study suggests clear habitat-dependent differences in the diversity patterns of invasive and native herbaceous plants within urban-rural ecotones (**Table 2**). Invasive herbaceous plants in roadside habitat exhibited the highest levels of species, functional, and phylogenetic diversity (**Figures 2A–F**). Roads act as major corridors for biological invasions, where the combination of frequent human disturbance, strong propagule dispersal, sparse canopy cover, and the removal of native vegetation collectively promotes the accumulation of alien seed banks and creates favourable conditions for invasive plant establishment and expansion (Buonopane et al., 2013; Lemke et al., 2019). Relative to other habitats, invasive herbaceous plant diversity was markedly lower in forest edge/understory, depression wetland, and landscape green space. In forest edge/understory, strong canopy shading, intense belowground competition, and relatively low disturbance impose pronounced environmental filtering, constraining the establishment of light-demanding invasive species (Zefferman et al., 2015; Dillon et al., 2018). Hydrological stress caused by excessive moisture and periodic inundation in depression wetlands allows only a small number of hydrophilic invasive species to become dominant, resulting in simplified invasive plant assemblages (Hunter and DeBerry, 2023). In the present study, *Alternanthera philoxeroides* and *Bidens alba* were the most commonly observed invasive species in depression wetlands. In landscape green space, frequent mowing and intensive management practices promote relatively stable vegetation structure and soil conditions, which reduces opportunities for invasive herbaceous plants to colonize and proliferate (Cangshuan et al., 2024).

However, functional dispersion of native herbaceous plants did not differ significantly among habitat types, indicating a relatively stable functional structure across urban-rural ecotones. This stability suggests that, through long-term environmental filtering, native plant communities have converged towards conservative and persistent combinations of functional traits, such that species capable of persisting across different habitats tend to share similar key traits (Cornwell and Ackerly, 2009; Garnier et al., 2016). Consequently, under the dynamic disturbance regimes characteristic of urban-rural ecotones, native plant communities may enhance ecosystem stability and adaptive capacity through the maintenance of a stable functional structure (De Bello et al., 2021).

In addition, Faith’s phylogenetic diversity and mean phylogenetic distance of native herbaceous plants were consistently higher than those of invasive herbaceous plants across all habitat types (**Figures 2E, F**). This pattern indicates that native communities exhibit a more phylogenetically dispersed structure, whereas invasive communities tend to be phylogenetically clustered, with relatively narrow family- and genus-level composition. This study recorded a total of 74 invasive herbaceous plant species, belonging to 26 families and 61 genera (**Supplementary Table 2**). Among them, Asteraceae was the most dominant family, accounting for 29.72% of all invasive species. This result aligns with earlier evidence identifying Asteraceae as one of the most widespread families among invasive plants globally (Yang et al., 2023). Representative genera included Erigeron, Bidens, and Ageratum, most of which originate from the Americas and are characterised by rapid growth and strong dispersal capacity (Huang et al., 2025b). In contrast, native herbaceous plant communities occupied deeper phylogenetic lineages, reflecting a stable pattern of coexistence shaped by long-term evolutionary history and niche differentiation (Tretyakova et al., 2021).

### 4.2 Coexistence of biotic acceptance and resistance effects

From an overall perspective, species richness of invasive herbaceous plants was positively correlated with native herbaceous plant species diversity within urban-rural ecotones, a pattern consistent with findings from urban forests reported by Kim et al. (2025). In environments characterised by intense human disturbance and high propagule pressure, native plant diversity does not necessarily translate into strong biotic resistance sensu Elton (Elton, 1958). Instead, such systems may better conform to the biotic acceptance hypothesis (Stohlgren et al., 2006), whereby habitats with higher heterogeneity offer a wider range of favourable conditions and a greater diversity of exploitable niches, thereby increasing opportunities for the establishment of alien species. The resource fluctuation hypothesis (Davis et al., 2000) further mechanistically explains this phenomenon, suggesting that disturbances can expose previously underutilised resources, such as water, light, and nutrients, which are readily captured by invasive species. Urban-rural ecotones are typically characterised by highly fragmented land use, frequent anthropogenic disturbance, and relatively low vegetation cover (Wandl and Magoni, 2017). These features promote spatially heterogeneous resource distribution and the persistent formation of niche vacancies that can be exploited by invasive species, thereby weakening the capacity of native plant communities to achieve complete resource pre–emption and reducing their overall resistance to invasion. Habitat-scale analysis confirmed that the positive correlation was mainly present in roadside habitats and at forest edges/understory (**Supplementary Figure 1**). This may be because forest understory biomass and species diversity are typically unsaturated (Gilbert and Lechowicz, 2005). In these habitats, relatively high environmental heterogeneity combined with recurrent disturbances generates a diversity of exploitable microhabitats, facilitating the establishment of invasive species (Bernes et al., 2017; Kotowska et al., 2022; Valadi et al., 2022; Gros et al., 2023).

In contrast, our results indicate that native herbaceous communities with high species, functional, and phylogenetic diversity are more effective at suppressing invasive herbaceous plant total cover and invasion intensity. This finding supports the framework of plant invasion resistance driven by 2D native diversity, which refers to the two-dimensional framework jointly composed of native biodiversity within the same trophic level (horizontal) and across trophic levels (vertical), emphasizing the importance of horizontal diversity across species, functional, and phylogenetic dimensions in enhancing community resistance to invasion (Huang et al., 2025a). This is consistent with the observation that native communities tend to exhibit greater phylogenetic dispersion compared to invasive communities, which are more phylogenetically clustered (**Figures 2E, F**). Consistent with this view, global-scale analyses have shown that high native phylogenetic and functional diversity provides an important ecological foundation for limiting invasion severity (Delavaux et al., 2023). Native communities characterised by greater functional differentiation and larger phylogenetic distances are able to exploit resources more completely, thereby strengthening niche complementarity and competitive exclusion, which in turn constrains further expansion of invasive species within already invaded communities. In the present study, these negative relationships were particularly pronounced in Abandoned land and Depression wetland. Abandoned land is typically associated with early successional stages and pulsed resource availability. Where native diversity is high, communities can rapidly develop vegetation cover following resource pulses, thereby reducing the frequency and persistence of niche vacancies available to invaders (Zhu et al., 2015; Bulleri et al., 2016). In depression wetland, stable hydrological conditions and elevated soil moisture favour the formation of dense aboveground cover and extensive belowground root systems by native herbaceous plants. These traits further restrict the resources and space available to invasive species, effectively reducing invasion cover (Byun et al., 2013; Petruzzella et al., 2018).

Native plant diversity can promote the richness of invasive species while suppressing their dual effects of coverage and invasion intensity, which may reflect the transition between species colonization and dominance establishment in two different dimensions. At the colonization level, abiotic environmental screening likely plays a dominant role, with habitat heterogeneity and high resource availability providing conditions for the coexistence of invasive and native species (Latombe et al., 2021). However, when establishing population dominance, biological interactions (especially competition) can become key limiting factors. Higher native plant diversity appears to reinforce competition for limiting resources by enhancing niche complementarity and resource priority effects (Tilman, 2004; Brown and Barney, 2021). Therefore, although diverse native communities may allow more invasive species to enter in the early stages, they have the potential to inhibit their transformation towards ecological dominance in terms of functionality (Levine et al., 2004). These findings highlight the importance of integrating multiple dimensions of biodiversity to better understand invasion outcomes.

### 4.3 Regulation of invasive herbaceous plants by environmental factors

Trampling intensity was positively associated with the species richness of invasive herbaceous plants. Human activities such as trampling and frequent passage can introduce alien propagules and simultaneously disturb the soil surface, creating microsites that facilitate seed dispersal and establishment, thereby increasing invasive species richness (Omar et al., 2018). In contrast, canopy cover were negatively correlated with invasive herbaceous plant species richness. Higher canopy cover likely limits light availability, a key factor for plant germination and growth (Gómez et al., 2022), and shaded environments therefore constrain the establishment and spread of light–demanding invasive species such as Chromolaena odorata and Wedelia trilobata. In contrast to patterns observed for species richness, proximity to buildings and proximity to water bodies were both positively correlated with the total cover of invasive herbaceous plants. Areas surrounding buildings are typically subject to more intense anthropogenic disturbance, which may create favourable conditions for the rapid colonization of invasive species (Godefroid and Ricotta, 2018; Liu et al., 2025a). Similarly, habitats adjacent to water bodies often experience higher soil moisture and nutrient inputs, creating favourable conditions for a small number of highly competitive and fast–expanding invasive herbaceous plants, particularly hygrophilous species such as Mikania micrantha and Chromolaena odorata, to rapidly capture resources and form dominant populations (Yue et al., 2019; Qin et al., 2024). As a result, a pronounced increase in invasive plant cover may drive community homogenisation, elevating ecological risks through dominance by a few aggressive invasive species.

In addition, relative humidity showed a significant positive relationship with invasive herbaceous plant total cover, but exhibited no significant association with species richness or invasion intensity. This contrasts with previous findings showing that higher humidity increased overall species abundance while reducing species richness across entire vegetation communities (He et al., 2024). This discrepancy likely reflects differences in the focal species pool. Invasive herbaceous plants, which are typically characterised by broad environmental tolerance and strong physiological resilience, are generally more responsive to humidity in terms of biomass accumulation rather than species turnover or diversity shifts (Chadha et al., 2019).

Finally, artificial removal was significantly negatively correlated with invasive herbaceous plant species richness, total cover, and invasion intensity. Direct removal of individuals and propagules through mowing or manual clearance effectively disrupts regeneration and dispersal processes of invasive herbaceous plants (Rudolph et al., 2017; Johnson et al., 2018), thereby substantially reducing invasion intensity. These results underscore that active management interventions may be among the most effective approaches for suppressing invasion pressure in urban-rural ecotones, particularly where frequent disturbance facilitates rapid regeneration, a conclusion that could be further tested through experimental or long-term management studies.

## 5 Conclusions

This study highlights the dual regulatory role of native herbaceous diversity in mediating biological invasions in urban-rural ecotones. By integrating invasive richness, cover, and invasion intensity, we found that biotic acceptance and biotic resistance coexist, contingent upon the assessment metric and habitat type. At the overall scale, intense disturbance and high propagule pressure facilitate initial colonization by increasing invasive species richness, whereas native diversity across species, functional, and phylogenetic dimensions effectively suppresses invasion severity by reducing invasive cover and intensity, indicating that functional complementarity and competitive exclusion in diverse communities limit the post-colonization expansion of invasive species. Further analyses revealed that habitat types and environmental factors played a critical role in shaping invasion patterns. These theoretical insights provide direct guidance for ecological restoration practices, shifting the management focus from the simple augmentation of native species richness to the construction of a multi-dimensional community defence system. We advocate a habitat-stratified management strategy that prioritizes the conservation of multidimensional diversity in less disturbed habitats to restore their natural resistance to invasions, while requiring continuous anthropogenic interventions in frequently disturbed environments. This study not only deepens our understanding of scale- and habitat-dependent invasion dynamics but also provides practical and ecologically sound guidance for the management of invasive plants in complex urban-rural ecotones.

## Data Availability

The data presented in the study are deposited in the Nutstore repository, accession link: https://www.jianguoyun.com/p/DcncoSgQsaGJDhiE250GIAA.

## References

[B1] Afonso L. Esler K. J. Gaertner M. Geerts S. (2020). “ Comparing invasive alien plant community composition between urban, peri-urban and rural areas; the city of Cape Town as a case study,” in Urban ecology (Amsterdam, Netherlands: Elsevier), 221–236. doi: 10.1016/B978-0-12-820730-7.00013-6, PMID:

[B2] Ariori C. Aiello-Lammens M. E. Silander J. (2017). Plant invasion along an urban-to-rural gradient in northeast connecticut. J. Urban. Ecol. 3, jux008. doi: 10.1093/jue/jux008. A. C.OMMAJ.R.X.X.X.

[B3] Bernes C. Bullock J. M. Jakobsson S. Rundlöf M. Verheyen K. Lindborg R. (2017). How are biodiversity and dispersal of species affected by the management of roadsides? A systematic map. Environ. Evid. 6, 24. doi: 10.1186/s13750-017-0103-1, PMID: 41796386

[B4] Boukita H. El Ammari M. Elwahab F. El Bahja F. Oudghiri M. Brhadda N. . (2025). Invasive plants in urban settings: a systematic review and bibliometric analysis of trends, gaps, and future implications. Trees For. People 22, 101051. doi: 10.1016/j.tfp.2025.101051, PMID: 41802445

[B5] Bradshaw C. J. A. Leroy B. Bellard C. Roiz D. Albert C. Fournier A. . (2016). Massive yet grossly underestimated global costs of invasive insects. Nat. Commun. 7, 12986. doi: 10.1038/ncomms12986, PMID: 27698460 PMC5059451

[B6] Brown B. L. Barney J. N. (2021). Rethinking biological invasions as a metacommunity problem. Front. Ecol. Evol. 8. doi: 10.3389/fevo.2020.584701, PMID: 41800340

[B7] Bulleri F. Benedetti-Cecchi L. Jaklin A. Iveša L. (2016). Linking disturbance and resistance to invasion via changes in biodiversity: a conceptual model and an experimental test on rocky reefs. Ecol. Evol. 6, 2010–2021. doi: 10.1002/ece3.1956, PMID: 27066222 PMC4767907

[B8] Buonopane M. Snider G. Kerns B. K. Doescher P. S. (2013). Complex restoration challenges: weeds, seeds, and roads in a forested wildland urban interface. For. Ecol. Manag. 295, 87–96. doi: 10.1016/j.foreco.2013.01.013, PMID: 41802445

[B9] Byun C. De Blois S. Brisson J. (2013). Plant functional group identity and diversity determine biotic resistance to invasion by an exotic grass. J. Ecol. 101, 128–139. doi: 10.1111/1365-2745.12016, PMID: 41803443

[B10] Cangshuan L. I. Qiongwen Z. Fei W. Hui L. I. U. (2024). Invasion resistance of herbaceous plant community: influence mechanism and design strategy. Landsc. Archit. 31, 79–85. doi: 10.3724/j.fjyl.202309150424, PMID: 41207781

[B11] Cao L. Wang G. Yang F. Li L. He R. (2024). Urbanization and plant diversity in urban fringes: differential responses across life forms. J. Environ. Manage. 371, 123151. doi: 10.1016/j.jenvman.2024.123151, PMID: 39509971

[B12] Chadha A. Florentine S. K. Chauhan B. S. Long B. Jayasundera M. (2019). Influence of soil moisture regimes on growth, photosynthetic capacity, leaf biochemistry and reproductive capabilities of the invasive agronomic weed; lactuca serriola. PloS One 14, e0218191. doi: 10.1371/journal.pone.0218191, PMID: 31251746 PMC6599151

[B13] Chatterjee S. Dewanji A. (2024). The distribution of invasive alien plant species in peri-urban areas: a case study from the city of Kolkata. Community Ecol. 25, 29–44. doi: 10.1007/s42974-023-00169-z, PMID: 41804457

[B14] Cheng C. Liu Z. Song W. Chen X. Zhang Z. Li B. . (2024). Biodiversity increases resistance of grasslands against plant invasions under multiple environmental changes. Nat. Commun. 15, 4506. doi: 10.1038/s41467-024-48876-z, PMID: 38802365 PMC11130343

[B15] Conradi T. Strobl K. Wurfer A. Kollmann J. (2015). Impacts of visitor trampling on the taxonomic and functional community structure of calcareous grassland. Appl. Veg. Sci. 18, 359–367. doi: 10.1111/avsc.12164, PMID: 41803443

[B16] Cornwell W. K. Ackerly D. D. (2009). Community assembly and shifts in plant trait distributions across an environmental gradient in coastal california. Ecol. Monogr. 79, 109–126. doi: 10.1890/07-1134.1

[B17] Dai W. Oduor A. M. O. Guo C. Quan Z. Li J. Zhao C. (2025). Distance from the road, habitat type and environmental factors predict distribution of invasive and native plant species in the above-ground vegetation and soil seedbanks. Divers. Distrib. 31, e70002. doi: 10.1111/ddi.70002, PMID: 41803443

[B18] Davis M. A. Grime J. P. Thompson K. (2000). Fluctuating resources in plant communities: a general theory of invasibility. J. Ecol. 88, 528–534. doi: 10.1046/j.1365-2745.2000.00473.x, PMID: 41717205

[B19] De Bello F. Lavorel S. Hallett L. M. Valencia E. Garnier E. Roscher C. . (2021). Functional trait effects on ecosystem stability: assembling the jigsaw puzzle. Trends Ecol. Evol. 36, 822–836. doi: 10.1016/j.tree.2021.05.001, PMID: 34088543

[B20] Delavaux C. S. Crowther T. W. Zohner C. M. Robmann N. M. Lauber T. van den Hoogen J. . (2023). Native diversity buffers against severity of non-native tree invasions. Nature 621, 773–781. doi: 10.1038/s41586-023-06440-7, PMID: 37612513 PMC10533391

[B21] Dillon W. W. Lieurance D. Hiatt D. T. Clay K. Flory S. L. Dillon W. W. . (2018). Native and invasive woody species differentially respond to forest edges and forest successional age. Forests 9. doi: 10.3390/f9070381, PMID: 41725453

[B22] Elton C. S. (1958). The ecology of invasions by animals and plants (London: Methuen & Co).

[B23] Ernst A. R. Barak R. S. Hipp A. L. Kramer A. T. Marx H. E. Larkin D. J. (2022). The invasion paradox dissolves when using phylogenetic and temporal perspectives. J. Ecol. 110, 443–456. doi: 10.1111/1365-2745.13812, PMID: 41803443

[B24] Ernst A. R. Larkin D. J. Kramer A. T. Glasenhardt M.-C. Hipp A. L. (2025). Diverse ecological strategies increase invasion resistance in an experimental grassland restoration. Ecol. Evol. 15, e71575. doi: 10.1002/ece3.71575, PMID: 40510631 PMC12158794

[B25] Fox J. Weisberg S. Price B. (2001). car: companion to applied regression. (Vienna: R Foundation for Statistical Computing). doi: 10.32614/CRAN.package.car, PMID:

[B26] Garnier E. Navas M.-L. Grigulis K. (2016). Plant functional diversity: organism traits, community structure, and ecosystem properties (Oxford, United Kingdom: Oxford University Press).

[B27] Gilbert B. Lechowicz M. J. (2005). Invasibility and abiotic gradients: the positive correlation between native and exotic plant diversity. Ecology 86, 1848–1855. doi: 10.1890/04-09997

[B28] Gioria M. Hulme P. E. Richardson D. M. Pyšek P. (2023). Why are invasive plants successful? Annu. Rev. Plant Biol. 74, 635–670. doi: 10.1146/annurev-arplant-070522-071021, PMID: 36750415

[B29] Godefroid S. Ricotta C. (2018). Alien plant species do have a clear preference for different land uses within urban environments. Urban. Ecosyst. 21, 1189–1198. doi: 10.1007/s11252-018-0792-4, PMID: 41804457

[B30] Gómez P. Espinoza S. Cuadros N. Goncalves E. Bustamante R. (2022). Light availability influences the invasion of teline monspessulana (L.) K. Koch in a temperate fragmented forest in central Chile. IForest. - Biogeosci. For. 15, 411. doi: 10.3832/ifor4026-015, PMID: 17959540

[B31] Gros C. Bulot A. Aviron S. Beaujouan V. Daniel H. (2023). Both management practices and landscape influence plant communities in urban grasslands. Front. Ecol. Evol. 11. doi: 10.3389/fevo.2023.1151913, PMID: 41800340

[B32] He R. Li L. Wang G. Cao L. Xiong G. Yang F. (2024). Plant diversity value of informal green spaces in tropical coastal urban areas: an empirical study of species, functional, and phylogenetic diversity. Sci. Tot. Environ. 955, 176741. doi: 10.1016/j.scitotenv.2024.176741, PMID: 39383971

[B33] Hou S. Tian C. Meng J. Liu C. Yao Z. (2023). The impact of urbanization on the distribution of spontaneous herbaceous plants in an ancient city: a pilot case study in jingzhou, China. Plants 12, 3353. doi: 10.3390/plants12193353, PMID: 37836093 PMC10574480

[B34] Huang Z. Lin M. Chen G. (2025b). Common agricultural weeds among alien invasive plants in China: species lists and their practical managing strategies. Heliyon 11, e41772. doi: 10.1016/j.heliyon.2025.e41772, PMID: 39882469 PMC11774770

[B35] Huang Q. Van Kleunen M. Liu Y. (2025a). Plant invasion resistance due to 2D native diversity. Trends Ecol. Evol. 40, 436–438. doi: 10.1016/j.tree.2025.02.009, PMID: 40102156

[B36] Hunter D. M. DeBerry D. A. (2023). Environmental drivers of plant invasion in wetland mitigation. Wetlands 43, 81. doi: 10.1007/s13157-023-01718-y, PMID: 41804457

[B37] Jauni M. Hyvönen T. (2012). Positive diversity–invasibility relationships across multiple scales in finnish agricultural habitats. Biol. Invasions. 14, 1379–1391. doi: 10.1007/s10530-011-0163-z, PMID: 41804457

[B38] Johnson A. L. Borowy D. Swan C. M. (2018). Land use history and seed dispersal drive divergent plant community assembly patterns in urban vacant lots. J. Appl. Ecol. 55, 451–460. doi: 10.1111/1365-2664.12958, PMID: 41803443

[B39] Jones B. McDermott S. (2018). Health impacts of invasive species through an altered natural environment: assessing air pollution sinks as a causal pathway. Environ. Resour. Econ. 71, 23–43. doi: 10.1007/s10640-017-0135-6, PMID: 41804457

[B40] Kembel S. W. Cowan P. D. Helmus M. R. Cornwell W. K. Morlon H. Ackerly D. D. . (2010). Picante: R tools for integrating phylogenies and ecology. Bioinformatics 26, 1463–1464. doi: 10.1093/bioinformatics/btq166, PMID: 20395285

[B41] Kim I. Sou H.-D. Cho H. Kim J. Oh J.-H. Park C.-R. (2025). Impact of urban forest structure, native species diversity, and vegetation community on invasive plant species richness. Urban. Ecosyst. 28, 6. doi: 10.1007/s11252-024-01658-3, PMID: 41804457

[B42] Kotowska D. Pärt T. Skórka P. Auffret A. G. Żmihorski M. (2022). Scale dependence of landscape heterogeneity effects on plant invasions. J. Appl. Ecol. 59, 1313–1323. doi: 10.1111/1365-2664.14143, PMID: 41803443

[B43] Kumar Rai P. Singh J. S. (2020). Invasive alien plant species: their impact on environment, ecosystem services and human health. Ecol. Indic. 111, 106020. doi: 10.1016/j.ecolind.2019.106020, PMID: 32372880 PMC7194640

[B44] Laliberte E. Legendre P. Shipley B. (2009). FD: measuring functional diversity (FD) from multiple traits, and other tools for functional ecology. (Vienna: R Foundation for Statistical Computing). doi: 10.32614/CRAN.package.FD, PMID:

[B45] Latombe G. Richardson D. M. McGeoch M. A. Altwegg R. Catford J. A. Chase J. M. . (2021). Mechanistic reconciliation of community and invasion ecology. Ecosphere. Wash. Dc. 12, e03359. doi: 10.1002/ecs2.3359, PMID: 34938590 PMC8647914

[B46] Lemke A. Kowarik I. Von Der Lippe M. (2019). How traffic facilitates population expansion of invasive species along roads: the case of common ragweed in Germany. J. Appl. Ecol. 56, 413–422. doi: 10.1111/1365-2664.13287, PMID: 41803443

[B47] Levine J. M. Adler P. B. Yelenik S. G. (2004). A meta-analysis of biotic resistance to exotic plant invasions. Ecol. Lett. 7, 975–989. doi: 10.1111/j.1461-0248.2004.00657.x, PMID: 41803443

[B48] Liu Y. Ren Y. Zhang H. Qiu D. Zhu Y. (2025a). Characteristics of invasive alien plants in different urban areas: the case of kunshan city, Jiangsu province, China. Front. Plant Sci. 16. doi: 10.3389/fpls.2025.1539457, PMID: 40190657 PMC11969225

[B49] Liu Z. Zhu B. Gao L. Wei C. Siemann E. Liu W. . (2025b). Native plant diversity provides resistance to invasion by an alien species in natural and experimental settings. Ecol. Lett. 28, e70137. doi: 10.1111/ele.70137, PMID: 40387160

[B50] Mao Q. Huang G. Buyantuev A. Wu J. Luo S. Ma K. (2014). Spatial heterogeneity of urban soils: the case of the Beijing metropolitan region, China. Ecol. Process. 3, 23. doi: 10.1186/s13717-014-0023-8, PMID: 41796386

[B51] Oksanen J. Simpson G. L. Blanchet F. G. Kindt R. Legendre P. Minchin P. R. . (2001). vegan: community ecology package. (Vienna: R Foundation for Statistical Computing). doi: 10.32614/CRAN.package.vegan, PMID:

[B52] Omar M. Al Sayed N. Barré K. Halwani J. Machon N. (2018). Drivers of the distribution of spontaneous plant communities and species within urban tree bases. Urban. For. Urban. Green. 35, 174–191. doi: 10.1016/j.ufug.2018.08.018, PMID: 41802445

[B53] Pek J. Wong O. Wong A. C. M. (2018). How to address non-normality: a taxonomy of approaches, reviewed, and illustrated. Front. Psychol. 9. doi: 10.3389/fpsyg.2018.02104, PMID: 30459683 PMC6232275

[B54] Petruzzella A. Manschot J. van Leeuwen C. H. A. Grutters B. M. C. Bakker E. S. (2018). Mechanisms of invasion resistance of aquatic plant communities. Front. Plant Sci. 9. doi: 10.3389/fpls.2018.00134, PMID: 29479363 PMC5811644

[B55] Potgieter L. J. Li D. Baiser B. Kühn I. Aronson M. F. J. Carboni M. . (2024). Cities shape the diversity and spread of nonnative species. Annu. Rev. Ecol. Evol. Syst. 55, 157–180. doi: 10.1146/annurev-ecolsys-102722-012749, PMID: 41139587

[B56] Qin W. Sun Y. Müller-Schärer H. Huang W. (2024). Responses of non-native and native plant species to fluctuations of water availability in a greenhouse experiment. Ecol. Evol. 14, e11692. doi: 10.1002/ece3.11692, PMID: 38983706 PMC11232050

[B57] Rudolph M. Velbert F. Schwenzfeier S. Kleinebecker T. Klaus V. H. (2017). Patterns and potentials of plant species richness in high- and low-maintenance urban grasslands. Appl. Veg. Sci. 20, 18–27. doi: 10.1111/avsc.12267, PMID: 41803443

[B58] Schmidt A. F. Finan C. (2018). Linear regression and the normality assumption. J. Clin. Epidemiol. 98, 146–151. doi: 10.1016/j.jclinepi.2017.12.006, PMID: 29258908

[B59] Seebens H. Bacher S. Blackburn T. M. Capinha C. Dawson W. Dullinger S. . (2021). Projecting the continental accumulation of alien species through to 2050. Glob. Change Biol. 27, 970–982. doi: 10.1111/gcb.15333, PMID: 33000893

[B60] Shi K. Naz M. Zhang C. Shao H. (2025). Aridity and grazing are associated with reduced trait complementarity and higher invasion intensity of solanum rostratum in native plant communities. Funct. Ecol. 39, 3255–3268. doi: 10.1111/1365-2435.70177, PMID: 41803443

[B61] Štajerová K. Šmilauer P. Brůna J. Pyšek P. (2017). Distribution of invasive plants in urban environment is strongly spatially structured. Landsc. Ecol. 32, 681–692. doi: 10.1007/s10980-016-0480-9, PMID: 41804457

[B62] Stohlgren T. J. Jarnevich C. Chong G. W. Evangelista P. H. (2006). Scale and plant invasions: A theory of biotic acceptance. Preslia 78, 405–426.

[B63] The Angiosperm Phylogeny Group Chase M. W. Christenhusz M. J. M. Fay M. F. Byng J. W. Judd W. S. . (2016). An update of the angiosperm phylogeny group classification for the orders and families of flowering plants: APG IV. Bot. J. Linn. Soc 181, 1–20. doi: 10.1111/boj.12385, PMID: 41803443

[B64] Tilman D. (2004). Niche tradeoffs, neutrality, and community structure: a stochastic theory of resource competition, invasion, and community assembly. Proc. Natl. Acad. Sci. U. S. A. 101, 10854. doi: 10.1073/pnas.0403458101, PMID: 15243158 PMC503710

[B65] Tretyakova A. S. Yakimov B. N. Kondratkov P. V. Grudanov N. Y. Cadotte M. W. (2021). Phylogenetic diversity of urban floras in the central urals. Front. Ecol. Evol. 9. doi: 10.3389/fevo.2021.663244, PMID: 41800340

[B66] Valadi G. Eshaghi Rad J. Khodakarami Y. Nemati Peykani M. Harper K. A. (2022). Edge influence on herbaceous plant species, diversity and soil properties in sparse oak forest fragments in Iran. J. Plant Ecol. 15, 413–424. doi: 10.1093/jpe/rtab090

[B67] Wandl A. Magoni M. (2017). Sustainable planning of peri-urban areas: introduction to the special issue. Plan. Pract. Res. 32, 1–3. doi: 10.1080/02697459.2017.1264191, PMID: 41799851

[B68] Wang C. Cheng H. Wang S. Wei M. Du D. (2021a). Plant community and the influence of plant taxonomic diversity on community stability and invasibility: a case study based on solidago canadensis L. Sci. Tot. Environ. 768, 144518. doi: 10.1016/j.scitotenv.2020.144518, PMID: 33454473

[B69] Wang G. Sun Y. Zhou M. Guan N. Wang Y. Jiang R. . (2021b). Effect of thinning intensity on understory herbaceous diversity and biomass in mixed coniferous and broad-leaved forests of Changbai Mountain. For. Ecosyst. 8, 53. doi: 10.1186/s40663-021-00331-x, PMID: 41796386

[B70] Wang T. Wang G. Innes J. L. Seely B. Chen B. (2017). ClimateAP: an application for dynamic local downscaling of historical and future climate data in Asia pacific. Front. Agric. Sci. Eng. 4, 448. doi: 10.15302/J-FASE-2017172

[B71] Xie J. Li X. Chung L. C. H. Webster C. J. (2024). Effects of land surface temperatures on vegetation phenology along urban–rural local climate zone gradients. Landsc. Ecol. 39, 62. doi: 10.1007/s10980-024-01856-6, PMID: 41804457

[B72] Xing F. Zhou J. Wang F. (2012). Inventory of plant species diversity in hainan (Wuhan: Huazhong University of Science and Technology Press).

[B73] Yang X. (2013). Checklist of plants of hainan (Beijing: Science Press).

[B74] Yang W. Sun S. Wang N. Fan P. You C. Wang R. . (2023). Dynamics of the distribution of invasive alien plants (asteraceae) in China under climate change. Sci. Tot. Environ. 903, 166260. doi: 10.1016/j.scitotenv.2023.166260, PMID: 37579809

[B75] Yue M. Yu H. Li W. Yin A. Cui Y. Tian X. (2019). Flooding with shallow water promotes the invasiveness of mikania micrantha. Ecol. Evol. 9, 9177–9184. doi: 10.1002/ece3.5465, PMID: 31463014 PMC6706175

[B76] Zefferman E. Stevens J. T. Charles G. K. Dunbar-Irwin M. Emam T. Fick S. . (2015). Plant communities in harsh sites are less invaded: a summary of observations and proposed explanations. AoB. Plants 7, plv056. doi: 10.1093/aobpla/plv056, PMID: 26002746 PMC4497477

[B77] Zhu D. H. Wang P. Zhang W. Z. Yuan Y. Li B. Wang J. (2015). Sampling and complementarity effects of plant diversity on resource use increases the invasion resistance of communities. PloS One 10, e0141559. doi: 10.1371/journal.pone.0141559, PMID: 26556713 PMC4640883

