# Supplementary material for: The dual role of native plant diversity in shaping plant invasions: scale and habitat dependence in urban-rural ecotones
Source: Front Plant Sci. 2026 Mar 13;17:1786551. doi: 10.3389/fpls.2026.1786551 (PMC13021677; doi:10.3389/fpls.2026.1786551)
Supplement: Supplementary file 1 [file DataSheet1.docx]

Supplementary Material

# Supplementary Tables

**Table S1.** Functional Characteristic Types of Herbaceous Plants.

| Order | Functional Traits | Variable type | Type of Function Traits | Source |
| --- | --- | --- | --- | --- |
| 1 | Life cycle | Categorical variable | 1. Annual/Biennial; 2. Perennial; 3. Other (variable/mixed life history) | Chinese Colored Weed Illustrated Book |
| 2 | Dormancy type | Categorical variable | 1. Summer annual herb; 2. Winter annual herb; 3. Perennial underground bud herb; 4. Perennial ground-germinating herb; 5. Perennial aboveground budding herb | Chinese Colored Weed Illustrated Book |
| 3 | Growth form | Categorical variable | 1. Branching; 2. Erect; 3. Vine; 4. Prostrate; 5. Rosette; 6. Tufted; 7. Stage-rosette; 8. Pseudo-rosette | Chinese Colored Weed Illustrated Book |
| 4 | Reproductive strategy | Categorical variable | 1. Sexual reproduction; 2. Asexual reproduction | Chinese Colored Weed Illustrated Book |
| 5 | Dispersal mechanism | Categorical variable | 1. Wind/Water dispersal; 2. Zoochory; 3. Autochory; 4. Gravity dispersal | Chinese Colored Weed Illustrated Book |
| 6 | Fruit type | Categorical variable | 1. Indehiscent fruit; 2. Dehiscent fruit; 3. Fleshy fruit | Chinese Colored Weed Illustrated Book |
| 7 | Phenological period | Categorical variable | 1. Short (< 3 months); 2. Medium (3~6 months); 3. Long (> 6 months) | Chinese Colored Weed Illustrated Book |

**Table S2.** Family and Genus Composition of Herbaceous Plants.

| Family | Genus | Species |  | Family | Genus | Species |
| --- | --- | --- | --- | --- | --- | --- |
| Asteraceae | 31 | 37 |  | Nyctaginaceae | 2 | 2 |
| Poaceae | 26 | 34 |  | Urticaceae | 2 | 2 |
| Amaranthaceae | 7 | 14 |  | Vitaceae | 2 | 2 |
| Cyperaceae | 6 | 14 |  | Araliaceae | 1 | 2 |
| Fabaceae | 11 | 11 |  | Lygodiaceae | 1 | 2 |
| Rubiaceae | 5 | 11 |  | Oxalidaceae | 1 | 2 |
| Euphorbiaceae | 5 | 8 |  | Portulacaceae | 1 | 2 |
| Polygonaceae | 3 | 8 |  | Adoxaceae | 1 | 1 |
| Convolvulaceae | 3 | 6 |  | Orchidaceae | 1 | 1 |
| Apiaceae | 4 | 4 |  | Phyllanthaceae | 1 | 1 |
| Araceae | 4 | 4 |  | Talinaceae | 1 | 1 |
| Plantaginaceae | 4 | 4 |  | Sapindaceae | 1 | 1 |
| Malvaceae | 4 | 4 |  | Ophioglossaceae | 1 | 1 |
| Solanaceae | 3 | 4 |  | Cleomaceae | 1 | 1 |
| Pteridaceae | 2 | 4 |  | Boraginaceae | 1 | 1 |
| Commelinaceae | 2 | 4 |  | Piperaceae | 1 | 1 |
| Brassicaceae | 3 | 3 |  | Acoraceae | 1 | 1 |
| Lamiaceae | 3 | 3 |  | Passifloraceae | 1 | 1 |
| Asparagaceae | 3 | 3 |  | Mazaceae | 1 | 1 |
| Cucurbitaceae | 3 | 3 |  | Thelypteridaceae | 1 | 1 |
| Verbenaceae | 3 | 3 |  | Menispermaceae | 1 | 1 |
| Onagraceae | 1 | 3 |  | Pontederiaceae | 1 | 1 |
| Linderniaceae | 2 | 2 |  | Pandanaceae | 1 | 1 |
| Acanthaceae | 2 | 2 |  | Aristolochiaceae | 1 | 1 |
| Amaryllidaceae | 2 | 2 |  | Marsileaceae | 1 | 1 |
| Molluginaceae | 2 | 2 |  |  |  |  |

**Table S3.** Results of one-way ANOVA testing the effects of habitat type on species, functional, and phylogenetic diversity indices of native and invasive herbaceous plants.

| **Diversity Indicators** | **Herbaceous Plant Type** | **F** | **P** |
| --- | --- | --- | --- |
| Species richness | Native herbs | 4.782 | 0.000277 |
|  | Invasive herbs | 10.392 | 1.59e-09 |
| Shannon-Wiener index | Native herbs | 4.106 | 0.00115 |
|  | Invasive herbs | 9.959 | 4.03e-09 |
| Functional richness index | Native herbs | 4.402 | 0.000627 |
|  | Invasive herbs | 10.658 | 8.97e-10 |
| Functional divergence index | Native herbs | 0.733 | 0.599 |
|  | Invasive herbs | 5.679 | 4.1e-05 |
| Phylogenetic diversity | Native herbs | 3.582 | 0.0034 |
|  | Invasive herbs | 9.192 | 2.11e-08 |
| Mean phylogenetic distance | Native herbs | 3.014 | 0.0111 |
|  | Invasive herbs | 3.394 | 0.005 |
| Coverage | Native herbs | 4.782 | 0.000277 |
|  | Invasive herbs | 7.227 | 1.47e-06 |

# Supplementary Figures


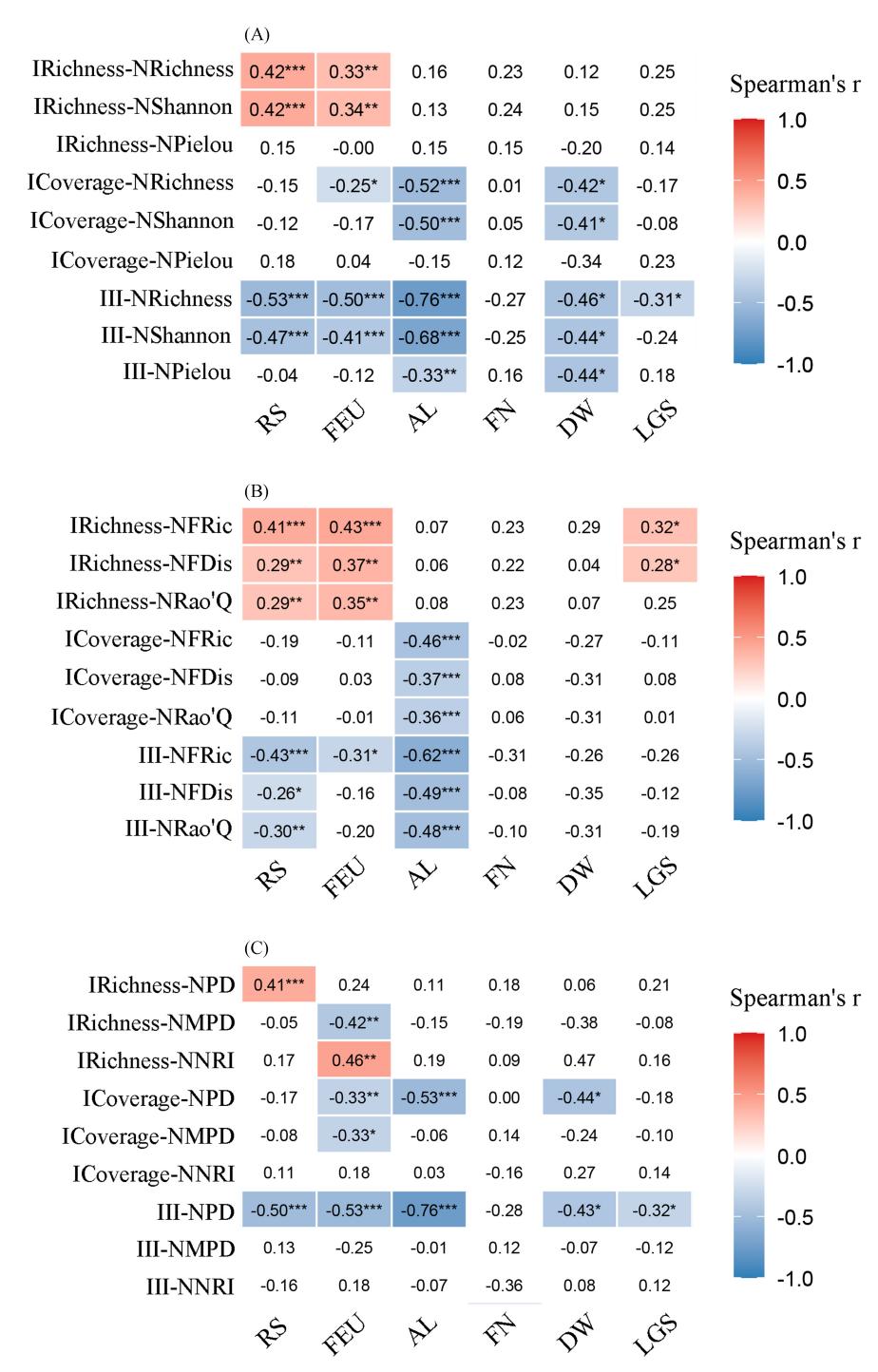


**Figure S1.** Heatmaps illustrating the Spearman correlations between invasive species richness, coverage, and invasion intensity indices and native herbaceous plant (A) species diversity, (B) functional diversity, and (C) phylogenetic diversity in different habitat types. Note: Values in the cells represent Spearman's correlation coefficients (r), and asterisks indicate significance levels (*** P < 0.001, ** P < 0.01, * P < 0.05). Red color denotes positive correlations, blue color denotes negative correlations, with darker shades indicating stronger correlations; white indicates non-significant correlations. The abbreviations are defined as follows: RS: Roadside habitat; FEU: Forest edge/understory; AL: Abandoned land; FN: Farmland/nursery; DW: Depression wetland; LGS: Landscape green space.
